# Ziv-aflibercept plus pembrolizumab in patients with advanced melanoma resistant to anti-PD-1 treatment

**DOI:** 10.1007/s00262-023-03593-2

**Published:** 2024-01-18

**Authors:** Joanna Baginska, Allison Nau, Ilana Gomez Diaz, Anita Giobbie-Hurder, Jason Weirather, Juliana Vergara, Charlotte Abrecht, Margaret Hallisey, Jenna Dennis, Mariano Severgnini, Julia Huezo, Isabella Marciello, Osama Rahma, Michael Manos, Andrew S. Brohl, Philippe L. Bedard, Daniel J. Renouf, Elad Sharon, Howard Streicher, Patrick A. Ott, Elizabeth I. Buchbinder, F. Stephen Hodi

**Affiliations:** 1 Department of Medical Oncology, Center for Immuno-Oncology, Dana-Farber Cancer Institute, Boston, MA USA; 2 Department of Data Science, Dana-Farber Cancer Institute, Boston, MA USA; 3 Sarcoma Department and Cutaneous Oncology, Moffitt Cancer Center, Tampa, FL USA; 4 Division of Medical Oncology and Hematology, Princess Margaret Cancer Centre, University Health Network, Toronto, ON Canada; 5 Cancer and Department of Medicine, University of British Columbia, Vancouver, BC Canada; 6 Cancer Therapy Evaluation Program, National Cancer Institute, Bethesda, MD USA

**Keywords:** Ziv-aflibercept, Immune checkpoint inhibition, Melanoma, Pembrolizumab, Clinical trials, Immune monitoring

## Abstract

**Background:**

Vascular endothelial growth factor is associated with reduced immune response and impaired anti-tumor activity. Combining antiangiogenic agents with immune checkpoint inhibition can overcome this immune suppression and enhance treatment efficacy.

**Methods:**

This study investigated the combination of ziv-aflibercept anti-angiogenic therapy with pembrolizumab in patients with advanced melanoma resistant to anti-PD-1 treatment. Baseline and on-treatment plasma and PBMC samples were analyzed by multiplex protein assay and mass cytometry, respectively.

**Results:**

In this Phase 1B study (NCT02298959), ten patients with advanced PD-1-resistant melanoma were treated with a combination of ziv-aflibercept (at 2–4 mg/kg) plus pembrolizumab (at 2 mg/kg), administered intravenously every 2 weeks. Two patients (20%) achieved a partial response, and two patients (20%) experienced stable disease (SD) as the best response. The two responders had mucosal melanoma, while both patients with SD had ocular melanoma. The combination therapy demonstrated clinical activity and acceptable safety, despite the occurrence of adverse events. Changes in plasma analytes such as platelet-derived growth factor and PD-L1 were explored, indicating potential alterations in myeloid cell function. Higher levels of circulating CXCL10 in non-responding patients may reflect pro-tumor activity. Specific subsets of γδ T cells were associated with poor clinical outcomes, suggesting impaired γδ T-cell function in non-responding patients.

**Conclusions:**

Although limited by sample size and follow-up, these findings highlight the potential of the combination of ziv-aflibercept antiangiogenic therapy with pembrolizumab in patients with advanced melanoma resistant to anti-PD-1 treatment and the need for further research to improve outcomes in anti-PD-1-resistant melanoma.

**Trial registration number:**

NCT02298959.

**Supplementary Information:**

The online version contains supplementary material available at 10.1007/s00262-023-03593-2.

## Introduction

Vascular endothelial growth factor (VEGF) is produced at high levels by tumor cells or stroma [1] and is associated with an increase in tumor angiogenesis. VEGF also has immunosuppressive properties and contributes to tumor metastasis by reducing the activity of antigen-presenting cells through the inhibition of dendritic cell maturation from hematopoietic progenitor cells [2]. High VEGF levels have been associated with CD8 + T-cell exhaustion [3], an increase in regulatory T-cell frequencies (Treg) [4], and outcomes from immune checkpoint inhibition (ICI) [5, 6]. Thus, combined therapy with immune checkpoint and angiogenesis inhibitors has been postulated as a promising strategy for the treatment of solid cancers [7].

The angiogenic inhibitor ziv-aflibercept, a soluble decoy of VEGF receptors 1 and 2, serves as a trap for circulating VEGF A and B. Ziv-aflibercept offers advantages over other anti-angiogenic agents such as TKIs and the VEGF-A inhibitor bevacizumab due to its increased affinity to both VEGF-A and VEGF-B [8, 9]. Ziv-aflibercept in combination with 5-fluorouracil, leucovorin, irinotecan-(FOLFIRI) is indicated for patients with metastatic colorectal cancer (mCRC) that is resistant to or has progressed following an oxaliplatin-containing regimen. Ziv-aflibercept improved survival in a phase III study of second-line treatment for metastatic colorectal cancer when combined with 5-fluorouracil, folinic acid, and irinotecan following progression with oxaliplatin [10]. Ziv-aflibercept was also evaluated for clinical activity in non-small cell lung cancer [11] and hormone-refractory prostate cancer [12].

Pembrolizumab, a monoclonal antibody against programmed cell death protein 1 (PD-1), has been tested in combination with anti-angiogenic agents in several clinical trials. When given in combination with a VEGF kinase inhibitor, pembrolizumab was found to modulate anti-tumor immune responses, resulting in improved outcomes for patients with metastatic renal cell carcinoma compared to tyrosine kinase inhibitor (TKI) alone [13, 14].

We previously reported favorable clinical outcomes in patients with a number of solid tumors who were treated with ziv-aflibercept (at 2–4 mg/kg) plus pembrolizumab (at 2 mg/kg) administered intravenously every 2 weeks [15]. The combination regimen was found to be safe, with no reported dose-limiting toxicities. Responses primarily occurred in cancers known to be responsive to ICI such as melanoma. Radiographic response correlated with activated tumor infiltrating CD8 T cells expressing CD8^+^PD1^+^, high CD40L expression, and increased peripheral memory CD8 T cells.

Following the previous work, we present here safety and efficacy data from an expansion cohort of 10 patients with advanced, anti-PD-1-resistant melanoma treated with the previously established combination regimen ziv-aflibercept and pembrolizumab. The primary objective of this multicenter, phase IB, open-label follow-up study was to determine safety and efficacy in this expansion cohort. Endpoints included overall survival and progression-free survival. Exploratory objectives included association of cytokine and immune cell profiles as well as immune cell activation and maturation changes with clinical response. We hypothesized that ziv-aflibercept would induce anti-tumor effects via its anti-angiogenic activity, and that combined with pembrolizumab could attenuate resistance to PD-1 inhibitors and improve clinical outcomes rate in advanced melanoma patients.

## Methods

### Study design and treatment

The study was sponsored by the Cancer Therapy Evaluation Program at the National Cancer Institute (NCI) and carried out at Dana-Farber Cancer Center (Boston, Massachusetts, USA), University Health Network-Princess Margaret Cancer Centre (Toronto, Ontario, Canada), Moffitt Cancer Center (Tampa, Florida, USA), and BC Cancer Center Agency-Vancouver Cancer Center (Vancouver, British Columbia, Canada).

The following were key eligibility criteria: Eastern Cooperative Oncology performance status (ECOG-PS) of 0 or 1; two prior lines of systemic therapy for metastatic disease; anticipated life expectancy of at least 6 months; and measurable disease according to Response Evaluation Criteria in Solid Tumors (RECIST) V.1.1.

Key exclusion criteria included prior chemotherapy, targeted small molecule therapy, or radiotherapy within 4 weeks prior to entering the study or inadequate recovery from adverse events due to agents administered more than 4 weeks earlier; new onset of immunodeficiency; immunosuppressive therapy within 7 days; monoclonal antibody therapy within 4 weeks; other systemic treatment for active autoimmune disease; high risk of gastrointestinal or pulmonary bleeding; inflammatory bowel disease, diverticulitis, pulmonary embolism, or uncontrolled thrombo-embolic event within 3 months history of peptic ulcer disease, erosive esophagitis, or gastritis; ulcerated skin lesions; active anticoagulation therapy with warfarin; blood pressure > 150/100 mmHg; and known or progressive brain metastases. There were no requirements listed in the inclusion/exclusion criteria about prior treatments for patients with BRAF-mutated disease.

### Safety and dose-limiting toxicity (DLT) assessment

Adverse events (AEs) were assessed using the NCI Common Terminology Criteria for Adverse Events (CTCAE) V.5.0. Our data did not collect whether an adverse event was immune-related. Dose-limiting toxicity (DLT) was classified as any of the following treatment-related AEs occurring within the first 4 weeks of therapy: unexpected grade ≥ 3 AEs likely attributable to treatment, ≥ 3 AEs that do not improve with or without intervention within 7 days of onset, eye pain grade ≥ 2, grade 3 hypertension that does not improve with appropriate medical intervention within 14 days, urine protein:creatinine ratio > 3.5 g or > 2 g protein in 24-h urine, two delays of treatment (not related to scheduling non-adherence) each lasting > 10 days within 4 cycles of the treatment, or arterial thromboembolic event.

### Clinical assessments

Anti-tumor activity was assessed based on RECIST V.1.1. Tumor assessments of all patients included a computed tomography (CT) scan of the chest, abdomen, and pelvis, as well as head magnetic resonance imaging (MRI) within 4 weeks of starting treatment. Tumor assessments were performed at baseline and every 12 weeks thereafter, and follow-up scans were obtained 6–8 weeks after initial documentation of objective response. Tumors were assessed based on RECIST V.1.1.

### Statistical analysis

The primary objective of this study was to determine the safety and toxicity rate of treatment with ziv-aflibercept plus pembrolizumab. All patients who received at least one dose of the combination treatment have safety data presented. The proportions of patients with adverse events grade 3 or higher are presented with 90% exact binomial confidence intervals (CIs).

Additionally, secondary endpoints, including overall response rate (ORR), progression-free survival (PFS), overall survival (OS), and time to disease progression (TTP), were assessed. Time-to-event endpoints (i.e., PFS and OS) are summarized and presented using the Kaplan–Meier product-limit method; 90% of CIs are based on log(-log(outcome)) methodology; medians are presented with 90% CIs.

The dose regimen was based on the dose escalation trial ziv-aflibercept 4 mg/kg and pembrolizumab 2 mg/kg intravenously every 2 weeks as previously reported [15]. The trial followed a two-stage Simon design, which allowed for early termination if efficacy was not detected or if the drug combination was too toxic for the indication. Designs were estimated to have one-sided type-I error rates of 0.10 and 85% power. The study would continue to the second stage if at least two patients responded, and the incidence of grade 3 or higher toxicities (i.e., possibly, probably, or definitely related) was less than 0.33. Once the trial progressed to the second stage, eight more patients would be enrolled. The treatment would be considered promising if 4 or more of the 18 melanoma patients responded to the pembrolizumab plus ziv-aflibercept combination.

For correlative studies, Wilcoxon rank-sum tests were used to compare pre-treatment Luminex measurements with a response or disease control categorized as complete response (CR), partial response (PR), or stable disease (SD). SD was defined using the RECIST method and was not linked to a duration. Longitudinal models, to assess relationships over time, were fit to log2 transform of marker expression; predictors in each model were response or disease control, time point, and their interaction. Each model was fit using a linear mixed model with an autoregressive covariance structure. Estimates of differences according to response or disease control, or of changes over time, were made using contrasts. Statistical analyses were performed using SAS V.9.4. Multiple comparisons adjustment for an FDR rate of 0.1 was based on the Benjamini–Hochberg procedure.

### Analysis of biomarkers and correlatives

Correlative analyses of peripheral blood mononuclear cells (PMBCs) and plasma were performed on samples from ten patients collected at pre-treatment and after 1 month on treatment. A panel of 19 cytokines and chemokines, including VEGFa, interleukin (IL)-1b, IL-6, IL-7, IL-10, IL-12, TNF-a, CX3CL3, CXCL10, and PD-L1, was analyzed using the Bio-Techne Luminex Assay Kit (Bio-Techne, Minneapolis, Minnesota).

Mass cytometry by time of flight (CyTOF(R)) was performed on PBMC samples at pre-treatment and 1 month following treatment initiation to investigate immunological changes during treatment. The antibody panel included phenotypic markers to characterize T cells, B cells, dendritic cells (DCs), monocytes, natural killer cells, checkpoints, and activation. Metal-tagged antibodies used for the mass cytometry panel are listed in Supplemental Table 4. All antibodies were used per the manufacturer’s recommendation. Mass cytometry data were normalized using Permessa software, and data were gated to live/single/CD45 positive subset using FlowJo software. Mass cytometry data were analyzed using CATALYST and diffcyt. Mass cytometry p-values were adjusted using the Benjamini and Hochberg procedure; adjusted *p *values less than *α* = 0.1 were considered significant.

## Results

### Patients

Here, we present the results for a melanoma expansion-cohort of a phase 1B multicenter, open-label study, in which the efficacy and safety of treatment with a ziv-aflibercept plus pembrolizumab in patients with advanced PD-1-resistant melanoma, renal cancer, and sarcoma were examined. Eighteen patients were enrolled across the three cohorts. In the melanoma cohort, ten patients with anti-PD-1-resistant advanced melanoma enrolled at four centers in the US and Canada between February 2019 and May 2020 received combination treatment with ziv-aflibercept and pembrolizumab. The mean follow-up time was 20.6 months. Patient demographics are summarized in Table 1. Most patients were Caucasian (80%), identified as female (70%), and the median age was 58 (range 39–87). All patients had an ECOG performance status of 0 or 1. Among the ten patients with melanoma, four had non-cutaneous disease, including two cases of mucosal melanoma and two cases of ocular melanoma. Four of the 10 patients had a non-cutaneous primary (two mucosal and two ocular). Four patients (40%) had received at least one previous line of chemotherapy and four patients (40%) had undergone radiation (Supp. Table 1).

**Table 1:** Baseline demographic and patient characteristics

Characteristic	Clinical benefit	
	Yes (*n* = 4)	No (*n* = 6)
Median age (years)	60.5 (range)	58.0 (range)
Median weight (kg)	93.1 (range)	70.7 (range)
Median BMI	28.3 (range)	28.1 (range)

	All		CB		NCB	
	*n*	%	*n*	%	*n*	%
Female	7	70.0	1	25.0	6	100.0
Male	3	30.0	3	75.0	–	–
*Race*						
Unknown	2	20.0	–	–	2	33.3
White	8	80.0	4	100.0	4	66.7
*Ethnicity*						
Not Hispanic/Latino	9	90.0	3	75.0	6	100.0
Unknown	1	10.0	1	25.0	–	–
*ECOG performance status*						
0	5	50.0	1	25.0	4	66.7
1	5	50.0	3	75.0	2	33.3
*Primary site of disease*						
Anus	2	20.0	1	25.0	1	16.7
Eye, globe	1	10.0	1	25.0	–	–
Melanoma (eye)	1	10.0	1	25.0	–	–
Nasopharynx	1	10.0	1	25.0	–	–
Shoulder	1	10.0	–	–	1	16.7
Sinuses	1	10.0	–	–	1	16.7
Skin	1	10.0	–	–	1	16.7
Temple	1	10.0	–	–	1	16.7
Thigh	1	10.0	–	–	1	16.7
*Disease stage*						
Stage III	1	10.0	1	25.0	–	–
Stage IIIB	1	10.0	–	–	1	16.7
Stage IV	8	80.0	3	75.0	5	83.3
*Prior therapy*						
Chemotherapy	3	30.0	2	50.0	1	16.67
Immunotherapy	10	100.0	4	100.0	6	100.0
Radiation	4	40.0	1	25.0	3	50.0
Surgery	10	100.0	4	100.0	6	100.0

BMI body mass index, *ECOG* Eastern Cooperative Oncology Group, *NCB* no clinical benefit, and *CB* clinical benefit

Patients received between 3 and 53 cycles of the outlined treatment scheme (see Methods) respective of the individual therapeutic plan and response. Outcome data were collected for all patients until study data lock on January 31, 2023. At that point, all 10 patients (100%) had stopped treatment. Seven patients discontinued treatment due to progressive disease, two due to treatment-related adverse events (TRAEs). One patient completed treatment protocol after 53 cycles despite early PD, which was allowed per protocol to account for possible pseudoprogression. One of the two patients who discontinued treatment due to TRAEs experienced grade 4 encephalitis and grade 4 meningitis after 3 cycles.

### Safety and toxicity

All 10 patients with melanoma enrolled on the trial experienced one or more TRAE (Supp. Table 2). The most common TRAEs included hypertension (80%), headache (50%), and fatigue (50%). Grade 3 or 4 TRAEs were reported for 5 of the 10 patients (90% CI 22–78%). Grade 3 TRAEs occurred in four patients and included fatigue in 1 patient (10%), neck pain in 1 patient (10%), headache in 1 patient (10%), and hypertension in 4 patients (40%). There were two grade 4 TRAEs: hypertension and encephalitis classified as definitively related to therapy. In this cohort, safety was comparable to the original safety profile, as 50% of melanoma patients reported ≥ grade 3 AEs compared to 58% ≥ grade 3 AEs in the initial study [15]. Due to the combination of efficacy and safety specified by the trial design to continue to the second stage, the trial did not to continue to stage 2.

### Efficacy

Two patients (20%) achieved partial response (PR) and two patients (20%) experienced stable disease (SD) as best response. The two responders had mucosal melanoma. Both patients with SD had ocular melanoma. The duration of response was 8.3 and 2.7 months, respectively; duration of SD was 1.8 and 0.8 months, respectively. The 3-month response rate was 10% [1 of 10], and the 6-month response rate was 20% (2 of 10) with the first PR occurring at 2.8 months and the second PR occurring at 5.5 months. For subsequent analyses, the six patients with reported progressive disease (PD) are referred to as patients with no clinical benefit (NCB). Median overall survival (OS) was 13.2 months (90% CI 7.3–20.4), and median progression-free survival (PFS) was 3.0 months (90% CI 2.6–8.2) (Supp. Figure 1).

### Association of circulating cytokines and clinical outcome

To interrogate circulating soluble immune markers for patients with either CB versus NCB, we analyzed steady-state and longitudinal differences in immune responses in serum samples collected at two timepoints: baseline prior to treatment (pre-treatment) and approximately 1 month after treatment onset (post-treatment) using Luminex Flexmap 3D® assay. Analyte expression levels in patient serum were compared between both response groups for each timepoint, respectively.

**Fig. 1:**
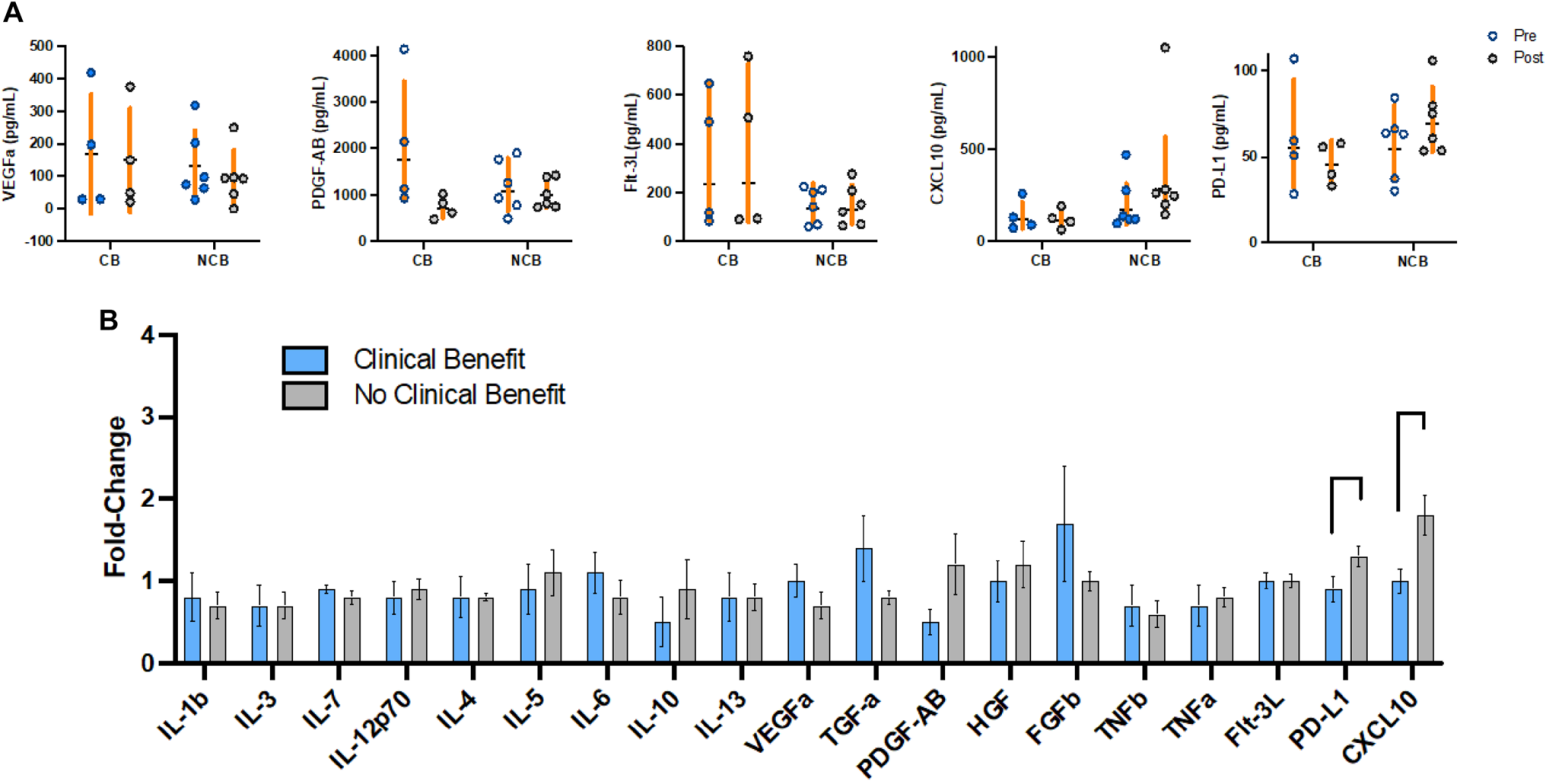
**a** Association between cytokine level and response to the pembrolizumab plus ziv-aflibercept combination. **b** Overall fold change of cytokine expression for CB and NCB over the course of treatment. Serum samples were collected from 10 patients during pre-treatment, and a panel of 19 cytokines and chemokines was analyzed using the Bio-Techne Luminex Assay Kit

There were no statistically significant differences in analyte expression levels between the response groups (Supp. Table 3) at baseline. To note, levels of VEGF-A were similar in both response groups at both timepoints (Fig. 1a), without any statistically significant differences. Baseline levels of PDGF-AB were numerically higher in CB patients compared to NCB patients at the baseline (*p* = 0.27), whereas post-treatment levels of PDGF-AB were reduced (*p* = 0.08; Fig. 1a) in CB patients, but not in NCB patients compared to baseline. Levels of FMS-like tyrosine kinase 3 ligand (FLT-3L), implicated in anti-tumor activity via mobilization of dendritic cells (DCs) [16], were higher in CB patients compared to NCB (Fig. 1a) both at baseline and on treatment. CXCL10, a chemokine known to negatively regulate recruitment of anti-tumor immune cells to tumor microenvironment (TME) [17], was elevated post-treatment in patients with NCB, but not in patients with CB (Fig. 1a). Post-treatment, there were slightly elevated levels of PD-L1 in NCB patients compared to the CB group (Fig. 1a). Programmed cell death ligand 1 (PD-L1) has been demonstrated to attenuate host immune response against various cancers [18].

Longitudinal cytokine expression levels between CB and NCB patients were compared for each of the 19 tested analytes (Fig. 1b). In NCB patients, there was a distinct difference in CXCL10 expression over time, with a median fold change of 1.9 (90% increase) compared to 0.9 (10% decrease) in CB (*p* = 0.04, NS after multiple hypothesis correction). Fold change expression of PD-L1, the principal ligand of PD-1, was higher in patients with NCB, with a median fold change of 1.3 (30% increase) compared to 0.9 (10% decrease) in the CB group (*p* = 0.04, NS after multiple hypothesis corrections).

### Association of immune cell populations abundance and clinical outcome

**Fig. 2:**
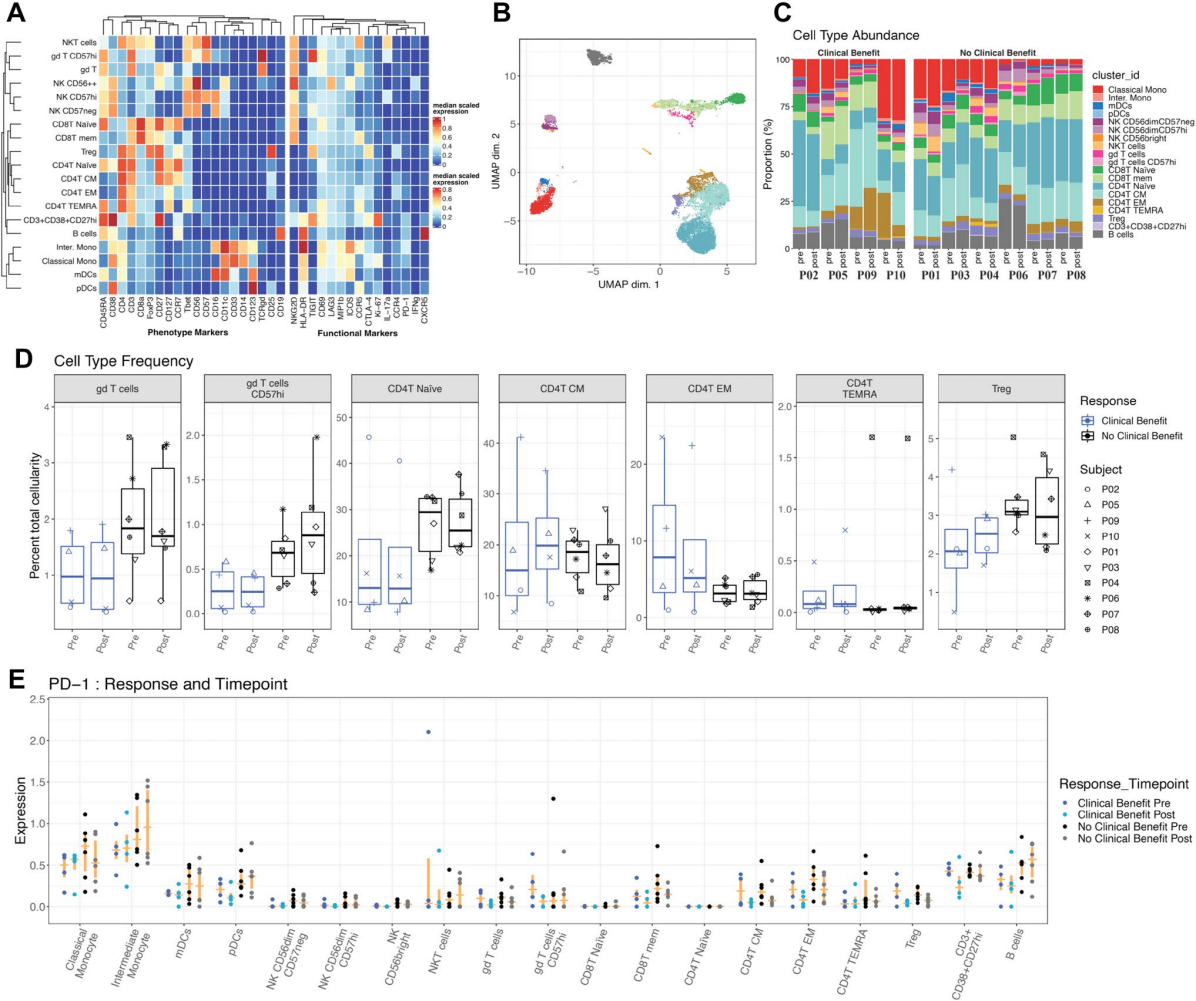
**a** Heatmap showing cluster population definitions. **b** UMAP dimensional reduction of mass cytometry data, color coded by cluster cell populations. **c** Stacked barplot showing frequency of each cell population in each patient sample. Legends for B and C shown in figure C. **d** Boxplot showing frequency of specific cell populations pre- and post-treatment for both response groups. **e** Arcsinh transformed expression of PD1. Median and interquartile range shown in orange

Immunophenotyping of PBMCs (Fig. 2a and b) collected pre- and post-treatment revealed changes in adaptive and innate immune phenotypes. We analyzed the proportional distribution of immune cell populations among all patients over the course of treatment (Fig. 2c and d, Supplemental Fig. 2). T cells of the γδ lineage are unconventional T cells whose function is not restricted to MHC-mediated antigen presentation. There were no significant changes in total γδ T cells frequencies in patients with NCB at pre- and post-treatment compared with CB; however in patients with NCB, γδ T cells expressing senescence marker CD57 were higher post-treatment (*p* = 0.03), these differences were not statistically significant (NS) after multiple hypothesis corrections (padj > 0.1). Analysis of αβ T-cell populations revealed increased frequencies of effector memory CD4^+^ T cells in CB patients at both timepoints compared to NCB (*p* = 0.029, padj = NS) (Fig. 2, Supplemental Fig. 2). No differences in B-cell nor natural killer T-cell (NKT) abundance between the two groups pre- and post-treatment were observed. At post-treatment, PD-1 was elevated in immune cell populations including intermediate monocytes, mDCs, pDCs, and B cells in NCB patients compared to CB. In addition, PD-1 expression was upregulated in CD4^+^ T effector memory cells in patients with NCB compared to patients with CB (*p* = 0.03, padj = NS Fig. 2e).

## Discussion

There is a substantial rationale for study of antiangiogenic therapy in combination with pembrolizumab in advanced melanoma resistant to anti-PD-1 treatment. Angiogenic factors, such as vascular endothelial growth factor (VEGF), play a crucial role in immune modulation, tumorigenesis, and the progression of tumors [16]. Elevated levels of VEGF have been associated with diminished anti-tumor immune response and resistance to immune checkpoint inhibition in the context of cancer treatment (a). Consequently, it is worth noting that a combination of antiangiogenic agents and immune checkpoint inhibitors (ICIs) may serve as a promising approach to overcome immune suppression caused by angiogenesis, a significant impediment in cancer therapy. In a previous study employing dose escalation, we determined a therapeutic dosage for the combined treatment of ziv-aflibercept, an antiangiogenic agent, and pembrolizumab in solid tumors, while maintaining an acceptable safety profile [12]. Overall, ziv-aflibercept in combination with pembrolizumab was well-tolerated and showed clinically meaningful activity. Toxicity for the combination was similar to observations previously reported with the combination. Interestingly, the combination therapy of ziv-aflibercept and pembrolizumab showed efficacy in anti-PD-1-resistant melanoma patients with ocular and mucosal disease, with a mean follow-up time of 20.6 months. However, the treatment was associated with adverse events, including a severe case of encephalitis and meningitis.

Efforts to define predictive biomarkers of ziv-aflibercept activity are needed to help enrich study populations to those patients most likely to experience clinical benefit from the addition of ziv-aflibercept. Monocytes and macrophages are recognized as significant contributors of PDGF, a tumorigenic factor, and potent angiogenesis regulator [16]. PDGF-AB, derived from platelets, has been documented to induce cellular proliferation [17]. In contrast with patients with CB who showed a decline in PDGF expression after treatment, patients with NCB did not exhibit a corresponding decrease. Our speculation is that anti-angiogenic therapy exerts an influence on myeloid cell populations, resulting in diminished levels of pro-tumorigenic PDGF. Additionally, PD-L1 has been implicated in tumor immune evasion and cancer progression in various cancers [18]. PD-L1 levels were reduced in CB patients compared to NCB after treatment, suggesting a benefit following antiangiogenic treatment due to changes in myeloid cell function and PD-L1 production. While intratumoral CXCL10 was identified as a potential favorable prognostic factor in metastatic melanoma [19], circulating CXCL10 has also been reported to have dual effects and cell growth as well as angiogenesis in cancer, depending on CXCR3 expression and other factors in the TME [20]. In this study, higher levels of secreted CXCL10 in NCB patients may be reflective of pro-tumor activity due to overexpression of CXCR3 in the TME.

In this study, patients who benefited clinically of ziv-aflibercept and pembrolizumab combination exhibited lower levels of CD57^−^ and CD57^+^ γδ T compared to NCB at both timepoints. T cells of the γδ lineage are unconventional T cells whose function is not restricted to MHC-mediated antigen presentation. While γδ T cells are generally described as pivotal immune cells in tumor immunosurveillance, there have been multiple reports of specific γδ T-cell subsets that are associated with poor clinical outcome in melanoma patients [21, 22, 23]. In addition to immunosuppressive cytokines released by regulatory γδ T cells, levels of pro-tumor cytokine IL-17a are elevated when TME is dominated by Vδ1 T cells. Furthermore, basal levels of IL-17a in melanoma patients were associated with poor clinical outcomes [22]. Although no difference in IL-17a expression of circulating total γδ T cells was seen in this study, there is a trend of higher IL-17a expression in various circulating cell populations in patients with NCB. Together, we suggest that local γδ T-cell subsets may be perturbed and may be negatively contributing to clinical outcomes in patients with NCB. It would require a more detailed analysis of tumor-derived γδ T cells to delineate this further. Notably, CD57^hi^ expressed by γδ T cells in NCB patients post-treatment can be considered a marker of inactivation or senescence. Considering the increased levels of γδ T cells CD57^hi^ following treatment in NCB, it further suggests a potential impairment in γδ T-cell function among patients with unfavorable clinical prognosis.

There are several limitations to this study, including small sample size, relatively short follow-up period for correlative studies, and limited availability of specimens for immune cell analysis in tumors. Although we observed a disease control rate similar to that of our previous study, the limited sample size in this study prevented us from drawing definitive conclusions regarding clinical efficacy. The expansion cohorts in Part 2 were each based on Simon two-stage designs with 10 patients in stages 1 and 8 additional patients in stage 2. In order to move to stage 2, there must have been two or more responses in the first 10 patients *and* the rate of grade 3 or higher TRAE had to be 0.33 or less. Even though there were two responders, the rate of TRAE was 50% (5 of 10 patients). Therefore, the study stopped at the end of the first stage because of the safety clause. It is important to emphasize that for the purpose of rigorous clinical reporting and to minimize the risk of false discoveries, we employed the Benjamini–Hochberg procedure to adjust *p *values.

Given the observed clinical benefits in patients who had previously undergone anti-PD-1 therapy, there is a strong rationale for further investigating the combination of immune checkpoint inhibition with anti-angiogenesis agents in individuals resistant to anti-PD-1 treatment. Our study findings provide important clinical insights, indicating that the combined treatment with angiogenesis blockade and PD-1 inhibition is both safe and effective in a subset of patients. Moreover, the results suggest that this combination therapy has the potential to modulate cytokine and immune cell population profiles, thereby shaping the anti-tumor immune response. These clinically significant findings highlight the relevance and potential of this approach in the management of patients with anti-PD-1 resistance, offering a promising avenue for future research and patient care. Improved understanding of checkpoint blockade combinations will lead to enhanced therapeutic options for patients with advanced melanoma resistant to anti-PD-1 treatment.

### Supplementary Information

Below is the link to the electronic supplementary material.

Supplementary file1 (PDF 8443 kb)

## References

[1] Mehnert JM, McCarthy MM, Jilaveanu L, Flaherty KT, Aziz S, Camp RL (2010). Quantitative expression of VEGF, VEGF-R1, VEGF-R2, and VEGF-R3 in melanoma tissue microarrays. Hum Pathol.

[2] Oyama T, Ran S, Ishida T, Nadaf S, Kerr L, Carbone DP (1998). Vascular endothelial growth factor affects dendritic cell maturation through the inhibition of nuclear factor-kappa B activation in hemopoietic progenitor cells. J Immunol.

[3] Voron T, Colussi O, Marcheteau E, Pernot S, Nizard M, Pointet AL (2015). VEGF-A modulates expression of inhibitory checkpoints on CD8+ T cells in tumors. J Exp Med.

[4] Wada J, Suzuki H, Fuchino R, Yamasaki A, Nagai S, Yanai K (2009). The contribution of vascular endothelial growth factor to the induction of regulatory T-cells in malignant effusions. Anticancer Res.

[5] Jianda Yuan JZ, Dong Z, Tandon S, Kuk D, Panageas KS, Wong P, Wu X, Naidoo J, Page DB, Wolchok JD, Stephen Hodi F (2014) Pretreatment serum VEGF is associated with clinical response and overall survival in advanced melanoma patients treated with ipilimumab 10.1186/2051-1426-1-S1-P247 PMC3991109 24778276

[6] Jenkins RW, Barbie DA, Flaherty KT (2018). Mechanisms of resistance to immune checkpoint inhibitors. Br J Cancer.

[7] Hodi FS, Lawrence D, Lezcano C, Wu X, Zhou J, Sasada T, Zeng W, Giobbie-Hurder A, Atkins MB, Ibrahim N, Friedlander P, Flaherty KT, Murphy GF, Rodig S, Velazquez EF, Mihm MC, Russell S, DiPiro PJ, Yap JT, Ramaiya N, Van den Abbeele AD, Gargano M, McDermott D (2014). Bevacizumab plus ipilimumab in patients with metastatic melanoma. Cancer Immunol Res.

[8] Hv C, Avd V, Hoekman K (2009). Tyrosine kinase inhibitors of VEGF receptors: clinical issues and remaining questions. FBL.

[9] Ciombor KK, Berlin J (2014). Aflibercept—a decoy VEGF receptor. Curr Oncol Rep.

[10] Van Cutsem E, Tabernero J, Lakomy R, Prenen H, Prausova J, Macarulla T (2012). Addition of aflibercept to fluorouracil, leucovorin, and irinotecan improves survival in a phase III randomized trial in patients with metastatic colorectal cancer previously treated with an oxaliplatin-based regimen. J Clin Oncol.

[11] Chen H, Modiano MR, Neal JW, Brahmer JR, Rigas JR, Jotte RM (2014). A phase II multicentre study of ziv-aflibercept in combination with cisplatin and pemetrexed in patients with previously untreated advanced/metastatic non-squamous non-small cell lung cancer. Br J Cancer.

[12] Tannock IF, Fizazi K, Ivanov S, Karlsson CT, Flechon A, Skoneczna I (2013). Aflibercept versus placebo in combination with docetaxel and prednisone for treatment of men with metastatic castration-resistant prostate cancer (VENICE): a phase 3, double-blind randomised trial. Lancet Oncol.

[13] Motzer R, Alekseev B, Rha SY, Porta C, Eto M, Powles T (2021). Lenvatinib plus Pembrolizumab or Everolimus for Advanced Renal Cell Carcinoma. N Engl J Med.

[14] Rini BI, Plimack ER, Stus V, Gafanov R, Hawkins R, Nosov D (2019). Pembrolizumab plus axitinib versus sunitinib for advanced renal-cell carcinoma. N Engl J Med.

[15] Rahma OE, Tyan K, Giobbie-Hurder A, Brohl AS, Bedard PL, Renouf DJ (2022). Phase IB study of ziv-aflibercept plus pembrolizumab in patients with advanced solid tumors. J Immunother Cancer.

[16] Cueto FJ, del Fresno C, Brandi P, Combes AJ, Hernández-García E, Sánchez-Paulete AR (2021). DNGR-1 limits Flt3L-mediated antitumor immunity by restraining tumor-infiltrating type I conventional dendritic cells. J Immunother Cancer.

[17] Brandt EF, Baues M, Wirtz TH, May J-N, Fischer P, Beckers A (2022). Chemokine CXCL10 modulates the tumor microenvironment of fibrosis-associated hepatocellular carcinoma. Int J Mol Sci.

[18] Han Y, Liu D, Li L (2020). PD-1/PD-L1 pathway: current researches in cancer. Am J Cancer Res.

[19] Reschke R, Yu J, Flood BA, Higgs EF, Hatogai K, Gajewski TF (2021). Immune cell and tumor cell-derived CXCL10 is indicative of immunotherapy response in metastatic melanoma. J Immunother Cancer.

[20] Liu M, Guo S, Stiles JK (2011). The emerging role of CXCL10 in cancer (Review). Oncol Lett.

[21] Pauza CD, Liou M-L, Lahusen T, Xiao L, Lapidus RG, Cairo C, et al (2018) Gamma delta T cell therapy for cancer: it is good to be local. Front Immunol 9 10.3389/fimmu.2018.01305 PMC6003257 29937769

[22] Girard P, Charles J, Cluzel C, Degeorges E, Manches O, Plumas J (2019). The features of circulating and tumor-infiltrating γδ T cells in melanoma patients display critical perturbations with prognostic impact on clinical outcome. Oncoimmunology.

[23] Wistuba-Hamprecht K, Martens A, Haehnel K, GeukesFoppen M, Yuan J, Postow MA (2016). Proportions of blood-borne Vδ1+ and Vδ2+ T-cells are associated with overall survival of melanoma patients treated with ipilimumab. Eur J Cancer.

