# Supplementary material for: Ziv-aflibercept plus pembrolizumab in patients with advanced melanoma resistant to anti-PD-1 treatment
Source: Cancer Immunol Immunother. 2024 Jan 18;73(1):17. doi: 10.1007/s00262-023-03593-2 (PMC10796592; doi:10.1007/s00262-023-03593-2)
Supplement: Supplementary file 1 — Supplementary file1 (PDF 8443 kb) [file 262_2023_3593_MOESM1_ESM.pdf]

Supplemental Figure 1

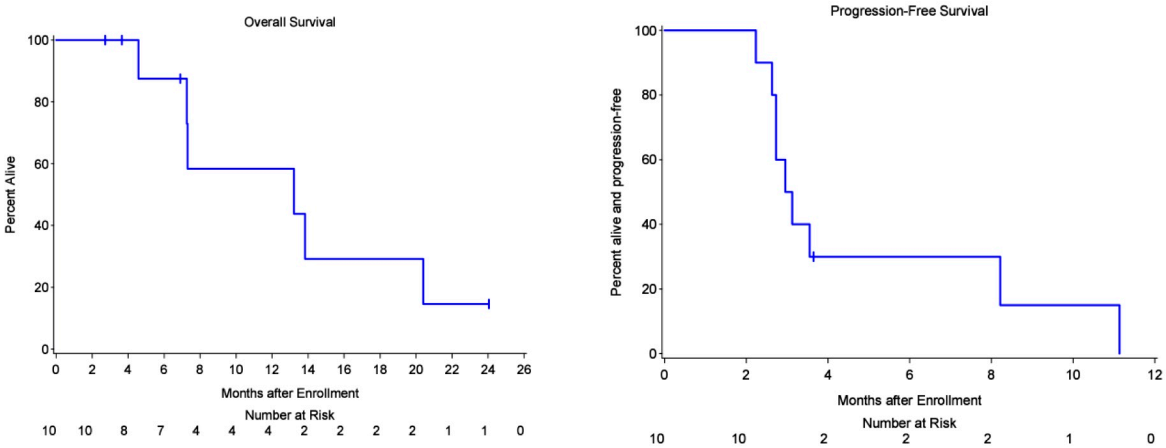

Kaplan-Meier estimates of overall and progression-free survival. Median overall survival (OS) was 13.2 months (90% CI: 7.3 – 20.4) and median progression-free survival (PFS) was 3.0 months (90% CI: 2.6-8.2)

<u>Supplemental Figure 2</u> Cell type frequencies, cellularity

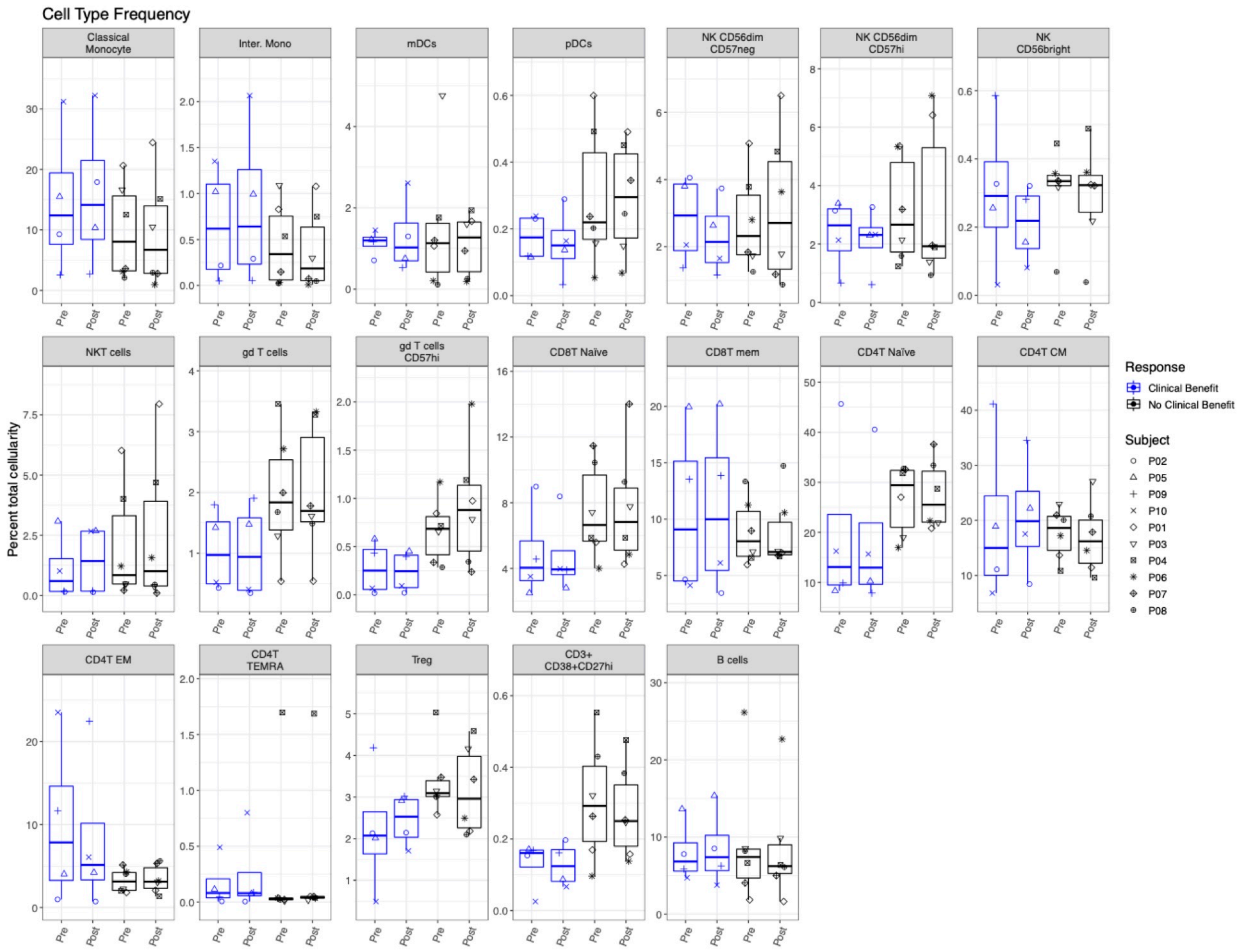

Changes in cell type frequencies of CB and NCB over the course of treatment

Supplemental Table 1 Prior systemic therapy

Prior Systemic Therapy	Number of Patients	No Clinical Benefit	Clinical Benefit
Ipi-Nivo	8	4	4
Nivo monotherapy	1	1	0
Pembro monotherapy	1	1	0
Ipi monotherapy	1	1	0
CMP-001	1	1	0
Temozolomide	1	0	1
Dabrafenib/Trametinib	1	0	1
Chemotherapy*	3	1	2

Abbreviations: Ipi, Ipilimumab; Nivo, Nivolumab; Pembro, Pembrolizumab

*Carboplatin/Paclitaxel, Cyclophosphamide/IRX-2, Cisplatin.

Supplemental Table 2 Treatment-related adverse events

System-Organ Class	Description	CTCAE Grade			
		1	2	3	4
		n=105	n=52	n=18	n=2
Blood and lymphatic system disorders	Anemia	2	-	-	-
	Eosinophilia	1	-	-	-
	Leukocytosis	-	-	1	-
Endocrine disorders	Hypothyroidism	-	1	-	-
Eye disorders	Decreased Close Visual Acuity	1	-	-	-
	Dry Eye	1	-	-	-
	Eye Pain	1	-	-	-
Gastrointestinal disorders	Abdominal Pain	-	1	1	-
	Constipation	2	-	-	-
	Diarrhea	4	-	-	-
	Dry Mouth	2	-	-	-
	Dyspepsia	-	1	-	-
	Dysphagia	1	-	-	-
	Gastrointestinal Pain	1	-	-	-
	Hemorrhoids	1	-	-	-
	Mucositis Oral	-	1	-	-
	Nausea	2	1	-	-
	Oral Dysesthesia	1	-	-	-
	Rectal Hemorrhage	1	-	-	-
	Vomiting	2	1	1	-
General disorders and administration site conditions	Chills	1	-	-	-
	Edema Limbs	2	-	-	-
	Fatigue	3	3	1	-
	Fever	-	-	1	-
	Flu Like Symptoms	1	-	-	-
	Infusion Related Reaction	1	-	-	-
	Pain	1	-	-	-
Infections and infestations	Encephalitis Infection	-	-	-	1
	Lung Infection	-	-	1	-
	Primary Tumor- Oropharynx	-	-	1	-
	Sinusitis	-	1	-	-
	Urinary Tract Infection	-	1	-	-
Injury, poisoning, and procedural complications	Fall	1	-	-	-
	Hernia	-	1	-	-
Investigations	Alanine Aminotransferase Increased	4	-	-	-
	Alkaline Phosphatase Increased	1	-	-	-
	Aspartate Aminotransferase Increased	3	1	-	-
	Blood Bilirubin Increased	1	-	-	-

	Blood Lactate Dehydrogenase Increased	1	-	-	-
	Creatinine Increased	3	-	-	-
	Increased Bun	1	-	-	-
	INR Increased	-	1	-	-
	Lipase Increased	2	1	-	-
	Lymphocyte Count Decreased	1	3	-	-
	Neutrophil Count Decreased	2	-	-	-
	Platelet Count Decreased	2	-	-	-
	Platelet Count Increased	1	-	-	-
	Serum Amylase Increased	1	1	-	-
	Weight Loss	3	-	-	-
Metabolism and nutrition disorders	Anorexia	-	3	-	-
	Dehydration	-	1	-	-
	Glucose Intolerance	1	-	-	-
	Hyperkalemia	2	-	-	-
	Hypoalbuminemia	-	3	-	-
	Hypocalcemia	-	1	-	-
	Hypokalemia	1	-	-	-
	Hyponatremia	5	-	-	-
	Hypophosphatemia	1	2	-	-
	Hypoproteinemia	1	-	-	-
Musculoskeletal and connective tissue disorders	Arthralgia	2	2	-	-
	Arthritis	1	-	-	-
	Avascular Necrosis	-	1	-	-
	Back Pain	1	-	-	-
	Flank Pain	-	-	1	-
	Generalized Muscle Weakness	1	-	-	-
	Left Chest Wall Pain	-	1	-	-
	Neck Pain	1	-	2	-
	Pain in Extremity	2	-	-	-
Neoplasms benign, malignant, and unspecified (incl cysts and polyps)	Tumor Pain	1	-	-	-
Nervous system disorders	Dizziness	2	-	-	-
	Dysesthesia	-	1	-	-
	Headache	2	4	2	-
	Paresthesia	-	1	-	-
Psychiatric disorders	Confusion	1	-	-	-
	Insomnia	1	1	-	-
Renal and urinary disorders	Hematuria	3	-	-	-
	Proteinuria	2	4	-	-
Respiratory, thoracic and mediastinal disorders	Allergic Rhinitis	-	1	-	-
	Cough	2	-	-	-
	Dyspnea	1	1	-	-
	Epistaxis	1	-	-	-
	Hoarseness	1	-	-	-
	Nasal Congestion	1	-	-	-

	Pulmonary Edema	-	1	-	-
	Rhinorrhea	1	-	-	-
	Sore Throat	1	-	-	-
Skin and subcutaneous tissue disorders	Alopecia	1	-	-	-
	Eczema- Right Palm	1	-	-	-
	Photosensitivity	1	-	-	-
	Pruritus	3	-	-	-
	Psoriasis	-	1	-	-
	Rash	1	-	-	-
	Rash Maculo-Papular	-	1	-	-
Vascular disorders	Hypertension	-	2	6	1
	Thromboembolic Event	-	1	-	-

Abbreviations: CTCAE, common terminology criteria for adverse events

<u>Supplemental Table 3</u> Cytokine measurements values

Analyte pg/mL	Clinical benefit	Non-clinical benefit	p-value
IL-1b	91.4 (1.0 – 679.9)	89.6 (1.0 – 566.7)	0.97
IL-3	889.8 (5.1 – 3909.6)	142.2 (1.0 – 1210.2)	0.61
IL-4	215.6 (10.6 – 668.0)	90.6 (19.6 – 467.4)	0.91
IL-5	9.4 (1.0 – 31.8)	6.3 (1.0 – 28.3)	0.81
IL-6	8.6 (2.2 – 24.7)	7.1 (1.1 – 16.7)	0.61
IL-7	24.7 (1.6 – 115.1)	10.0 (3.2 – 64.8)	0.99
IL-10	7.1 (1.0 – 22.5)	3.7 (1.0 – 13.5)	0.99
IL-12p70	1023.5 (30.5 – 3563.8)	308.6 (9.1 – 2694.0)	0.91
IL-13	38038.0 (27648.0 – 57338.0)	36280.0 (1.0 – 53411.0)	0.91
CXCL10	109.4 (71.9 – 258.8)	128.4 (97.6 – 470.4)	0.35
FGFb	35.4 (1.1 – 120.0)	5.2 (0.3 – 58.4)	0.76
Flt-3 ligand	303.3 (83.0 – 647.7)	170.4 (60.6 – 223.5)	0.48
HGF	273.2 (55.7 – 364.4)	187.1 (91.6 – 953.5)	0.91
PD-L1	55.1 (28.3 – 107.1)	63.5 (30.1 – 84.3)	0.76
PDGF-AB	1637.5 (936.1 – 4138.6)	1097.7 (489.3 – 1905.5)	0.27
TGFa	199.2 (1.6 – 432.1)	57 (6.7 – 164.2)	0.91
TNFa	64.4 (1.0 – 347.1)	32.5 (2.0 – 241.8)	0.99
TNFb	120.2 (1.0 – 555.8)	29.0 (1.0 – 335.3)	0.97
VEGFa	113.5 (29.1 – 418.6)	86.0 (27.8 – 318.0)	0.99

All data are presented in median (range) pg/mL.

Supplemental Table 4 Mass cytometry antibody panel

Metal	Marker	Clone
142Nd	CD19	HIB19
143Nd	CD45RA	HI100
144Nd	CD69	FN50
145Nd	CD4	RPA-T4
146Nd	CD8a	RPA-T8
147Sm	CD11c	Bu15
148Nd	ICOS	C398.4A
149Sm	CD56	NCAM16.2
150Nd	LAG3	11C3C65
151Eu	CD123	6H6
152Sm	TCRγδ	11F2
153Eu	CXCR5	RF8B2
154Sm	CD3	UCHT1
155Gd	CD27	L128
156Gd	CD14	HCD14
158Gd	CD33	WM53
159Tb	TIGIT	MBSA43
160Gd	MIP1b	D21-1351
161Dy	Tbet	4B10
162Dy	FoxP3	259D/C7
163Dy	CD57	HCD57
164Dy	IL-17a	N49-653
165Ho	IFNg	B27
166Er	NKG2D	ON72
167Er	CCR7	G043H7
168Er	Ki-67	B56
169Tm	CD25	2A3
170Er	CTLA-4	14D3
171Yb	CCR5	NP6G4
172Yb	CD38	HIT2
173Yb	HLA-DR	L243
174Yb	PD-1	EH12.2H7
175Lu	CCR4	L291H4
176Yb	CD127	A019D5
209Bi	CD16	3G8

Key Resources Table

REAGENT or RESOURCE	SOURCE	IDENTIFIER
Antibodies		
Anti-Human CD19 (HIB19)-142Nd—100 Tests (1:100)	Fluidigm	Cat#3142001B
Anti-Human CD45RA (HI100)-143Nd—100 Tests (1:100)	Fluidigm	Cat#3143006B
Anti-Human CD69 (FN50)-144Nd—100 Tests (1:100)	Fluidigm	Cat#3144018B
Anti-Human CD4 (RPA-T4)-145Nd—100 Tests (1:100)	Fluidigm	Cat#3145001B
Anti-Human CD8 (RPA-T8)-146Nd—100 Tests (1:100)	Fluidigm	Cat#3146001B
Anti-Human CD11c (Bu15)-147Sm—100 Tests (1:100)	Fluidigm	Cat#3147008B
Anti-CD278/ICOS (C398.4A)-148Nd—100 Tests (1:100)	Fluidigm	Cat#3148019B
Anti-Human CD56/NCAM (NCAM16.2)-149Sm—100 Tests (1:100)	Fluidigm	Cat#3149021B
Anti-Human CD223/LAG-3 (11C3C65)-150Nd—100 Tests (1:100)	Fluidigm	Cat#3150030B
Anti-Human CD123/IL-3R (6H6)-151Eu—100 Tests (1:100)	Fluidigm	Cat#3151001B
Anti-Human TCRgd (11F2)-152Sm—100 Tests (1:100)	Fluidigm	Cat#3152008B
Anti-Human CD185/CXCR5 (RF8B2)-153Eu—100 Tests (1:100)	Fluidigm	Cat#3153020B
Anti-Human CD3 (UCHT1)-154Sm—100 Tests (1:200)	Fluidigm	Cat#3154003B
Anti-Human CD27 (L128)-155Gd—100 Tests (1:100)	Fluidigm	Cat#3155001B
Anti-Human CD14 (HCD14)-156Gd—100 Tests (1:100)	Fluidigm	Cat#3156019B
Anti-Human CD33 (WM53)-158Gd—100 Tests (1:100)	Fluidigm	Cat#3158001B
Anti-Human TIGIT (MBSA43)-159Tb—100 Tests (1:100)	Fluidigm	Cat#3159038B
Anti-Human/Mouse MIP1beta (D21-1351)-160Gd—100 Tests (1:100)	Fluidigm	Cat#3160013B
Anti-Human/Mouse Tbet (4B10)-161Dy—100 Tests (1:100)	Fluidigm	Cat#3161014B
Anti-Human FoxP3 (259D/C7)-162Dy—50 Tests (1:100)	Fluidigm	Cat#3162024A
Anti-Human CD57 (HCD57)-163Dy—100 Tests (1:200)	Fluidigm	Cat#3163022B
Anti-Human IL-17A (N49-653)-164Dy—100 Tests (1:100)	Fluidigm	Cat#3164002B

Anti-Human IFNg (B27)-165Ho—100 Tests (1:100)	Fluidigm	Cat#3165002B
Anti-Human CD314/NKG2D (ON72)-166Er—100 Tests (1:100)	Fluidigm	Cat#3166016B
Anti-Human CD197/CCR7 (G043H7)-167Er—50 Tests (1:100)	Fluidigm	Cat#3167009A
Anti-Ki-67 (B56)-168Er—100 Tests (1:100)	Fluidigm	Cat#3168007B
Anti-Human CD25 (2A3)-169Tm—100 Tests (1:100)	Fluidigm	Cat#3169003B
Anti-Human CD152/CTLA-4 (14D3)-170Er—100 Tests (1:100)	Fluidigm	Cat#3170005B
Anti-Human CD195/CCR5 (NP-6G4)-171Yb—50 Tests (1:50)	Fluidigm	Cat#3171017A
Anti-Human CD38 (HIT2)-172Yb—100 Tests (1:100)	Fluidigm	Cat#3172007B
Anti-Human HLA-DR (L243)-173Yb—100 Tests (1:200)	Fluidigm	Cat#3173005B
Anti-Human CD279/PD-1 (EH12.2H7)-174Yb—100 Tests (1:100)	Fluidigm	Cat#3174020B
Anti-Human CD194/CCR4 (L291H4)-175Lu—50 Tests (1:50)	Fluidigm	Cat#3175035A
Anti-Human CD127/IL-7Ra (A019D5)-176Yb—100 Tests (1:100)	Fluidigm	Cat#3176004B
Anti-Human CD16 (3G8)-209Bi—100 Tests (1:100)	Fluidigm	Cat#3209002B
Chemicals, peptides, and recombinant proteins		
Ficoll-Paque™ Plus	Sigma-Aldrich	Cat#17144003
Dimethyl Sulfoxide (DMSO)	Fisher BioReagents	Cat#BP231-100
Gibco™ Phosphate-Buffered Saline (PBS)	Thermo Fisher Scientific	Cat#10010023
Antibiotic-Antimycotic (100×)	Gibco	Cat#15240096
Bovine Serum Albumin (BSA), 30% ± 2% in 0.85% NaCl	Sigma-Aldrich	Cat#A7284
Sodium Azide, 10% (w/v) solution in Ultra-Pure H_2O	Teknova	Cat#S0209
RPMI Medium 1640 [+] L-glutamine	Thermo Fisher Scientific	Cat#11875093
Gibco™ Premium Plus Fetal Bovine Serum, heat inactivated, qualified (FBS)	Thermo Fisher Scientific	Cat#10438026
UltraPure™ DNAse/RNAse-Free Distilled Water	Invitrogen	Cat#10977023
ViaStain™ AOPI Staining Solution	Nexcelom Biosciences	Cat#CS2-0106-5mL
Cell-ID™ Intercalator-103Rh—500 µM	Fluidigm	Cat#201103A
Cell-ID™ Intercalator-191/193Ir—125 µM	Fluidigm	Cat#201192B
Maxpar® Cell Acquisition Solution	Fluidigm	Cat#201237

Maxpar® Cell Staining Buffer	Fluidigm	Cat#201068
Maxpar® 10× Barcode Perm Buffer	Fluidigm	Cat#201057
EQ™ Four Element Calibration Beads	Fluidigm	Cat#201078
eBioscience™ Permeabilization Buffer (10×)	Invitrogen	Cat#00833356
eBioscience™ Fixation/Permeabilization Concentrate	Invitrogen	Cat#00512343
eBioscience™ Fixation/Permeabilization Diluent	Invitrogen	Cat#00522356
Human TruStain FcX™ (FcR Blocking Solution)	BioLegend	Cat#422302
Pierce™ 16% Formaldehyde Solution (w/v), Methanol-free	Thermo Fisher Scientific	Cat#28906
CyTOF® Tuning Solution, 250 mL	Fluidigm	Cat#201072
Critical commercial assays		
Cell-ID™ 20-Plex Pd Barcoding Kit	Fluidigm	Cat#201060
Biological samples		
Human PBMCs isolated from whole blood clinical samples (sex, age as required par study)	Any Supplier	N/A
Software and algorithms		
FlowJo v.10	Becton Dickinson & Company	FlowJo
GraphPad Prism v9.2	GraphPad Software, LLC	GraphhPad Prism
CyTOF Software v7.0.8493	Fluidigm	CyTOF Software
Other		
CoolCell™ LX Cell Freezing Vial Containers	Corning Inc.	Cat#07210001
15 mL Polypropylene Centrifuge Tubes	Corning Inc.	Cat#430052
50 mL Polypropylene Centrifuge Tubes	Corning Inc.	Cat#430829
Cellometer® SD100 Cell Counting Chamber Slides	Nexcelom Biosciences	CHT4-SD100-014
70 µm Cell Strainer, Polypropylene Frame	Biologix	Cat#15-1070
Eppendorf® 1.5 mL Safe-Lock Microcentrifuge Tubes, natural	Eppendorf	Cat#022363204
96-well Polystyrene V-Bottom Plate, untreated	Corning Inc.	Cat#3896
Polystyrene 96-well Microplate Corner Notch Lid	Corning Inc.	Cat#3930
Falcon® 5 mL Round Bottom Polypropylene Tubes (FACS tubes)	Corning Inc.	Cat#352063
Falcon® 5 mL Round Bottom Polystyrene Tubes with 35 µm Cell Strainer Snap Caps	Corning Inc.	Cat#352235
ThermoFisher Scientific™ Sorvall™ Legend™ XTR Refrigerated Centrifuge, 120VAC	Thermo Fisher Scientific	Cat#75004521

Fisherbrand™ Mini-Centrifuge 100–240 V, 50/6-Hz Universal Plug, Grey	Thermo Fisher Scientific	Cat#12-006-901
Fisher Scientific Digital Vortex Mixer	Thermo Fisher Scientific	Cat#0215370
Thermo Scientific™ Precision™ Circulating Water Bath	Thermo Fisher Scientific	Cat#TSCIR19
Cellometer® Auto 2000 Cell Viability Counter	Nexcelom Biosciences	Auto 2000
Fluidigm Helios™ Mass Cytometer	Fluidigm	N/A
Nebulizer for Helios™ Mass Cytometer	Fluidigm	Cat#107144
WB Injector for Helios™ Mass Cytometer	Fluidigm	Cat#107950

Complete RPMI

Reagent	Final concentration	Amount
RPMI Medium 1640 (1×)	n/a	500 mL
FBS	10%	50 mL
Antibiotic-Antimycotic (100×)	1%	5 mL
Total	n/a	555 mL

Store at 4°C for up to 2 weeks.

CyFACS

Reagent	Final concentration	Amount
PBS	n/a	500 mL
30% BSA	0.5%	8.3 mL
5% Sodium Azide	0.02%	2 mL
Total	n/a	510.3 mL

Store at 4°C for up to 6 weeks.

FoxP3 Fixation/Permeabilization Buffer

Reagent	Final concentration	Amount
Fixation/Permeabilization Concentrate	25%	8 mL
Fixation/Permeabilization Diluent	75%	32 mL
Total	n/a	40 mL
